# Population-level impact of a pulse oximetry remote monitoring programme on mortality and healthcare utilisation in the people with COVID-19 in England: a national analysis using a stepped wedge design

**DOI:** 10.1136/emermed-2022-212378

**Published:** 2022-04-13

**Authors:** Thomas Beaney, Jonathan Clarke, Ahmed Alboksmaty, Kelsey Flott, Aidan Fowler, Jonathan Benger, Paul P Aylin, Sarah Elkin, Ana Luisa Neves, Ara Darzi

**Affiliations:** 1 Department of Primary Care and Public Health, Imperial College London, London, UK; 2 Patient Safety Translational Research Centre, Institute of Global Health Innovation, Imperial College London, London, UK; 3 Centre for Mathematics of Precision Healthcare, Department of Mathematics, Imperial College London, London, UK; 4 NHS England and Improvement, London, UK; 5 NHS Digital, Leeds, UK; 6 Imperial College Healthcare NHS Trust, London, UK

**Keywords:** COVID-19

## Abstract

**Background:**

To identify the population-level impact of a national pulse oximetry remote monitoring programme for COVID-19 (COVID Oximetry @home (CO@h)) in England on mortality and health service use.

**Methods:**

We conducted a retrospective cohort study using a stepped wedge pre-implementation and post-implementation design, including all 106 Clinical Commissioning Groups (CCGs) in England implementing a local CO@h programme. All symptomatic people with a positive COVID-19 PCR test result from 1 October 2020 to 3 May 2021, and who were aged ≥65 years or identified as clinically extremely vulnerable were included. Care home residents were excluded. A pre-intervention period before implementation of the CO@h programme in each CCG was compared with a post-intervention period after implementation. Five outcome measures within 28 days of a positive COVID-19 test: (i) death from any cause; (ii) any ED attendance; (iii) any emergency hospital admission; (iv) critical care admission and (v) total length of hospital stay.

**Results:**

217 650 people were eligible and included in the analysis. Total enrolment onto the programme was low, with enrolment data received for only 5527 (2.5%) of the eligible population. The period of implementation of the programme was not associated with mortality or length of hospital stay. The period of implementation was associated with increased health service utilisation with a 12% increase in the odds of ED attendance (95% CI: 6% to 18%) and emergency hospital admission (95% CI: 5% to 20%) and a 24% increase in the odds of critical care admission in those admitted (95% CI: 5% to 47%). In a secondary analysis of CO@h sites with at least 10% or 20% of eligible people enrolled, there was no significant association with any outcome measure.

**Conclusion:**

At a population level, there was no association with mortality before and after the implementation period of the CO@h programme, and small increases in health service utilisation were observed. However, lower than expected enrolment is likely to have diluted the effects of the programme at a population level.

**Key messages**

**What is already known on this topic**

- The COVID Oximetry @home (CO@h) programme was implemented in November 2020 to provide pulse oximeters to people with confirmed or suspected COVID-19 infection to support self-monitoring.
- A pilot of the programme was identified as being a safe pathway for patients but the effectiveness of the programme remains unknown.

**What this study adds**

- Overall enrolment onto the programme in eligible people was low (2.5%).
- At a population level in England, there was no association with a change in mortality after implementation of the programme, and small increases in ED attendances and emergency hospital admissions.

**How this study might affect research, practice or policy**

- Our findings suggest the CO@h programme is a safe pathway for patients with COVID-19, but due to low total enrolment at a population level, further research is needed to identify whether the programme is effective at an individual level.

## Background

Since the start of the COVID-19 pandemic, asymptomatic (‘silent’) hypoxaemia has complicated the assessment and care of patients with COVID-19.^1^ Hypoxaemia has been shown to be an important predictor of mortality and the need for hospital admission in patients with COVID-19, yet those patients with asymptomatic hypoxaemia may be unaware of dangerously low blood oxygen saturations.^2 3^ Pulse oximetry allows patients and clinicians to regularly monitor a patient’s oxygen saturation and promptly initiate escalation of care should deterioration occur, such as triggering hospital assessment or admission.^1^ Health systems across the world introduced remote monitoring pathways, including the use of pulse oximetry, to support the care of patients with COVID-19 outside hospital.^4 5^

In November 2020, NHS England and Improvement introduced the COVID Oximetry @home (CO@h) programme, recommending that all Clinical Commissioning Groups (CCGs; responsible for local healthcare commissioning) in England provide services to monitor patients with a diagnosis of COVID-19 at home using pulse oximetry.^6^ The service built on local remote monitoring services provided by individual CCGs and hospital trusts earlier in the pandemic. CCGs were responsible for establishing services in their area, although these could be shared between CCGs, and more than one could operate within a single CCG.^7^ People enrolled were provided with a pulse oximeter and encouraged to record regular oxygen saturation readings with advice to call emergency services for readings of 92% or less, or to contact primary care services for readings of 93%–94%. There was no single model for CO@h, with differences across sites in how readings were recorded and reported (eg, via an app or via paper and telephone) and in the frequency of staff contact.^4 7 8^

Patients were eligible for the CO@h programme if they had symptomatic COVID-19 and were aged 65 years or older or at high risk from COVID-19, although some sites adopted broader eligibility criteria and criteria could vary over time.^7 9^ Additionally, clinical judgement could be applied to consider other individual risk factors,including pregnancy, learning disability and socioeconomic deprivation. The programme accepted patients from primary care, NHS Test and Trace, ambulance services and A&E departments, in contrast to ‘COVID-19 Virtual Wards’ which aimed to support discharge of patients with COVID-19 from hospital.^10^

The clinical effectiveness of the CO@h programme on mortality and secondary care utilisation was unknown. The primary aim of this analysis is to identify differences in mortality and use of healthcare services at a population level after implementation of the CO@h programme among eligible people. A secondary aim is to identify the impact of the programme in sites with a high total enrolment onto the programme among the eligible population.

## Methods

This study used a retrospective cohort of people eligible for the CO@h programme, comparing outcomes at a CCG level using a stepped wedge pre-implementation and post-implementation design. A population approach was chosen to reduce the impact of biases in patient selection which may occur at an individual level. Eligibility was defined as the population resident in England, with a positive COVID-19 PCR test result, who were symptomatic at the time of testing, from 1 October 2020 to 3 May 2021. Due to differing eligibility across sites and over time, and the role of clinical judgement, we selected for analysis the group of people who would have met the minimum eligibility criteria throughout: people aged 65 years or older, or those who were at high risk (see box 1). Those at high risk were identified through the NHS Digital Shielded Patient List as clinically extremely vulnerable (CEV).^9^ The conditions and risk factors determining CEV status are shown in the online supplemental appendix p3 and box A1 and). Care home residents were excluded from the analysis, as previous work has suggested significantly higher mortality in this group.^11^

10.1136/emermed-2022-212378.supp1 Supplementary data

Box 1 **Eligibility criteria for the COVID Oximetry @home programme**

- Diagnosed with COVID-19: either clinically or positive test result AND
- Symptomatic AND EITHER
- Aged 65 years or older OR
- Under 65 years and at higher risk from COVID-19 or where clinical judgement applied considering individual risk factors such as pregnancy, learning disability, caring responsibilities and/or deprivation. Pregnant women being referred to a COVID Oximetry @home service should also be asked to contact their maternity team for specific advice around pregnancy and COVID-19.

A lighter touch pathway should be available to any adult aged 18–64 years, that has tested positive and has not been double vaccinated. This pathway is fully self- managed and escalated.

Five outcomes were selected to capture impact on mortality and healthcare utilisation. Outcomes were defined as occurring within 28 days of the date of a positive COVID-19 test, for consistency with government-reported metrics^12^:

1. Death from any cause.
2. One or more A&E department attendances.
3. One or more emergency hospital admissions.
4. One or more critical care admissions (of those admitted to hospital).
5. Total hospital length of stay in days, of those admitted who did not die within 28 days.

### Data sources and processing

COVID-19 testing data were provided through the Second Generation Surveillance System,^13^ which collates positive COVID-19 test results conducted in laboratories across England. Data were available from 1 October 2020 to 3 May 2021. This analysis used PCR tests, with symptoms documented at the time of testing.^14^ Where more than one test was recorded, only the date of first test was used. Data on the number of patients enrolled onto the CO@h programme were submitted from CO@h sites via NHS Digital’s Strategic Data Collection Service.^15^ Primary care data were sourced from the General Practice Extraction Service Data for Pandemic Planning and Research (GDPPR).^16^ CEV status was sourced from NHS Digital’s Shielded Patient List linked to GDPPR. Hospital Episode Statistics (HES) data^17^ and the Emergency Care Data Set (ECDS)^18^ provided data on hospital admissions and ED (24-hour consultant led or specialist) attendances up to 31 May 2021. Data on registration of deaths were sourced from the Office for National Statistics, with data available up to 5 July 2021. Datasets were linked using a deidentified NHS patient ID.

The study population were assigned to the CCG they were resident in when the test was performed. Patient demographic data, including age, sex, ethnicity, lower layer super output area (LSOA) of residence were derived from GDPPR, or, if missing, from HES or ECDS. LSOA was linked to measures of socioeconomic deprivation based on deciles of the Index of Multiple Deprivation (IMD) 2019 for England.^19^ Data on care home residence, body mass index (BMI) and smoking status were available from GDPPR only. Information on 12 chronic conditions were included, extracted from GDPPR (online supplemental appendix). For demographics and chronic conditions, the most recent codes up to and including the date of a positive COVID-19 test were used to exclude those which may have resulted from COVID-19 infection. If no data were available prior to the date of a positive test for age, sex and ethnicity only, then the earliest data following the positive test was used. Full details of the datasets and cleaning approach are provided in the online supplemental appendix, with a link to the code lists.

### Statistical analysis

The pre-implementation and post-implementation periods were defined for each CO@h site, with implementation start dates for each site provided by NHS England @home. A stepped wedge design was used. All eligible people in each of the 106 CCGs in England before and after implementation of the CO@h programme were allocated to the control group and intervention group, respectively (irrespective of whether enrolment data were received for an individual). Two-level hierarchical regression models were run for each outcome, incorporating random intercepts for CCG. Logistic regression was used for the four binary end points and negative binomial regression models were used for the single continuous outcome (length of stay). Analyses of length of stay excluded patients who died within the 28-day time window.

To account for possible changes in the baseline risk of each outcome over time, the primary models for each outcome incorporated fixed effects for the month of positive COVID-19 test. To account for potential differences in the at-risk population before and after implementation, the primary models adjusted patient-level risk factors. Final covariates in each model included age category (years), sex, ethnicity, IMD score, BMI category, month of COVID-19 test, CEV status and clinical conditions. Intraclass correlation coefficients were calculated for each model. Sensitivity analyses were conducted to explore the robustness of results to adjustment for time and patient risk factors (online supplemental appendix).

A secondary analysis was performed on the subset of sites with a higher proportion of eligible people enrolled. Two thresholds were defined a priori, at 10% or more and 20% or more across the whole study period.

Analyses were conducted in the Big Data and Analytics Unit Secure Environment, Imperial College. Python V.3.9.5 and Pandas V.1.2.3 were used in data manipulation. Regression models were conducted in Stata V.17.0, using the *melogit* and *menbreg* commands.

### Patient and public involvement

Patients or the public were not involved in the design, conduct or reporting of our research.

## Results

**Figure 1:**
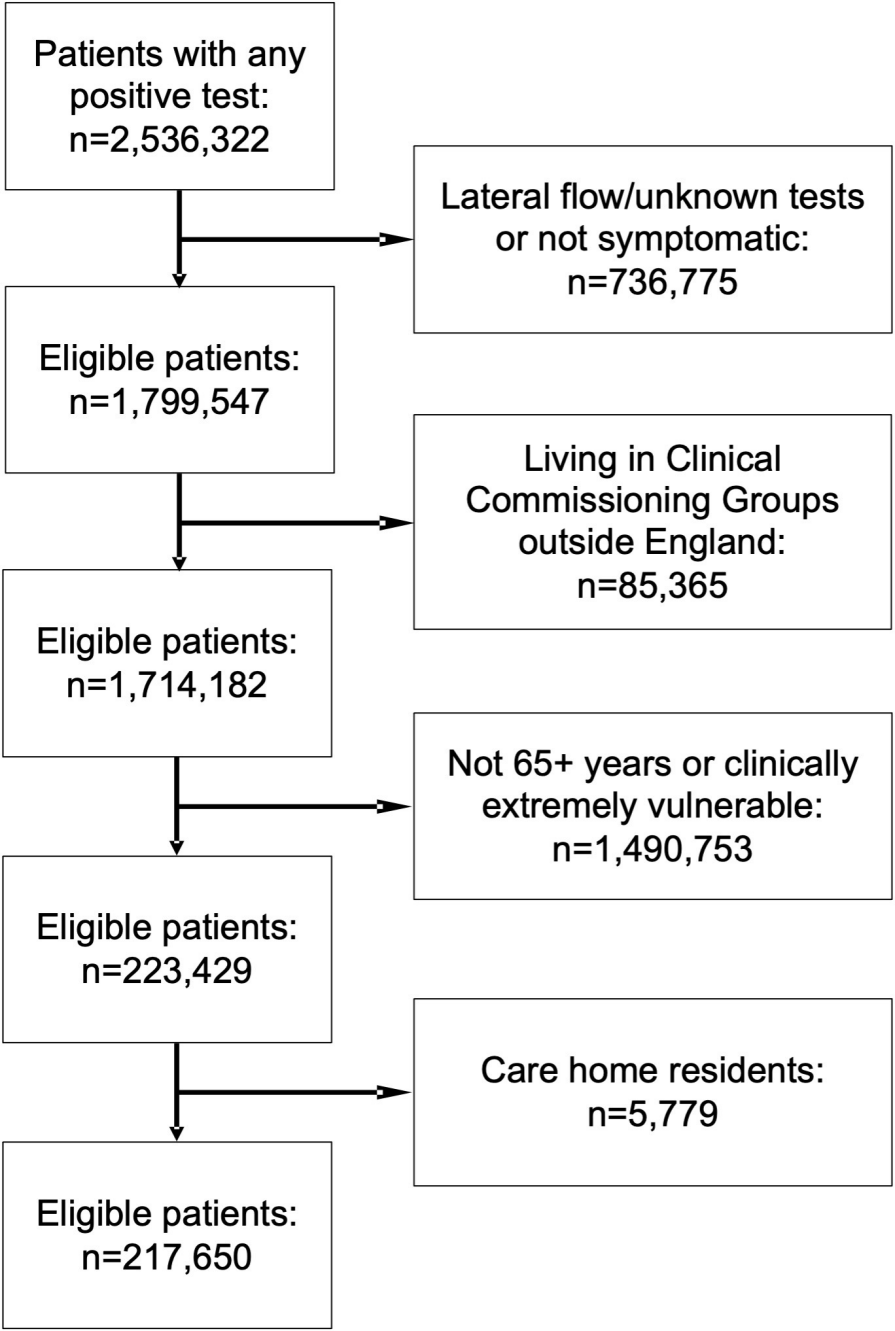
Flow diagram of eligibility criteria for the evaluation of the COVID Oximetry @home programme.

A total of 1 714 182 people resident in 106 CCGs in England had a positive PCR COVID-19 test between 1 October 2020 and 3 May 2021, and were symptomatic at the time of the test. A total of 223 429 (13.0%) were at least 65 years of age or CEV and were eligible for the analysis. Of these, 5779 (2.6%) were living in a care home and were excluded. A total of 217 650 people were included in the analysis (figure 1).

The mean (SD) age of participants was 62.4 (15.9) years, with 53.4% between 65 and 79 years. More women than men were enrolled (54.4% vs 43.9%). The majority were of white ethnicity (72.5%) and resident in the most socioeconomically deprived half of LSOAs in England (57.5%). More of the population were obese (39.4%) than overweight (33.6%) or had a healthy weight (20.8%). Just over half (54.9%) were never smokers. Hypertension (40.6%), diabetes (31.4%; type 1 and type 2) and chronic respiratory disease (29.3%) were the most common comorbidities. A total of 5616 (2.6%) of the study population died within 28 days of a positive COVID-19 test, 19.9% attended ED at least once, 12.2% were admitted at least once and of those admitted, 16.1% required critical care. There were significant differences in distributions of most of the predictor and outcome variables in the eligible population before and after implementation in each site (table 1). In the pre-implementation period, 77.6% were of white ethnic backgrounds, compared with 69.8% in the period after implementation. The percentage of deaths, ED attendances and admissions within 28 days from positive test were all significantly higher in the post-implementation period.

**Table 1:** Characteristics of people eligible for the CO@h programme from 1 October 2020 to 3 May 2021, before and after implementation at each site

	Total		Pre-implementation		Post-implementation		P value for difference
	Number	Percentage (%)	Number	Percentage (%)	Number	Percentage (%)	
**Age category (years) and clinically extremely vulnerable status**							
18–49 and CEV	48 502	22.3	15 364	20.5	33 138	23.2	
50–64 and CEV	31 538	14.5	10 319	13.8	21 219	14.9	
65–79 and not CEV	100 582	46.2	36 401	48.6	64 181	45.0	<0.001
65–79 and CEV	15 736	7.2	5726	7.6	10 010	7.0	
80+ and not CEV	14 145	6.5	4875	6.5	9270	6.5	
80+ and CEV	7147	3.3	2215	3.0	4932	3.5	
**Sex**							
Female	118 311	54.4	39 701	53.0	78 610	55.1	
Male	95 655	43.9	33 896	45.3	61 759	43.3	<0.001
Missing	3684	1.7	1303	1.7	2381	1.7	
**Ethnicity**							
White	157 815	72.5	58 112	77.6	99 703	69.8	
Asian/Asian British	36 482	16.8	10 725	14.3	25 757	18.0	
Black/African/Caribbean/Black British	8386	3.9	1631	2.2	6755	4.7	<0.001
Mixed/Multiple ethnic groups	2550	1.2	685	0.9	1865	1.3	
Other ethnic group	4548	2.1	1076	1.4	3472	2.4	
Missing	7869	3.6	2671	3.6	5198	3.6	
**Index of multiple deprivation decile**							
1 (most deprived)	28 245	13.0	11 697	15.6	16 548	11.6	
2	27 425	12.6	9207	12.3	18 218	12.8	
3	25 417	11.7	8038	10.7	17 379	12.2	
4	22 911	10.5	7233	9.7	15 678	11.0	<0.001
5	21 104	9.7	6884	9.2	14 220	10.0	
6	20 075	9.2	6510	8.7	13 565	9.5	
7	19 413	8.9	6691	8.9	12 722	8.9	
8	19 030	8.7	6839	9.1	12 191	8.5	
9	18 235	8.4	6380	8.5	11 855	8.3	
10 (least deprived)	15 746	7.2	5409	7.2	10 337	7.2	
Missing	49	0.0	12	0.0	37	0.0	
**Body mass index**							
Underweight	2370	1.1	724	1.0	1646	1.2	
Healthy weight	45 180	20.8	15 181	20.3	29 999	21.0	
Overweight	73 239	33.6	25 648	34.2	47 591	33.3	<0.001
Obese	85 834	39.4	29 770	39.7	56 064	39.3	
Missing	11 027	5.1	3577	4.8	7450	5.2	
**Smoking status**							
Never smoker	119 431	54.9	39 901	53.3	79 530	55.7	
Ex-smoker	66 438	30.5	24 770	33.1	41 668	29.2	<0.001
Current smoker	27 714	12.7	8862	11.8	18 852	13.2	
Missing	4067	1.9	1367	1.8	2700	1.9	
**Comorbidities**							
Hypertension	88 358	40.6	30 548	40.8	57 810	40.5	0.194
Chronic cardiac disease	35 300	16.2	12 482	16.7	22 818	16.0	<0.001
Chronic kidney disease	3346	1.5	1070	1.4	2276	1.6	0.003
Chronic respiratory disease	63 790	29.3	22 514	30.1	41 276	28.9	<0.001
Dementia	2763	1.3	790	1.1	1973	1.4	<0.001
Diabetes	68 444	31.4	21 558	28.8	46 886	32.8	<0.001
Chronic neurological disease (including epilepsy)	9330	4.3	3004	4.0	6326	4.4	<0.001
Learning disability	1496	0.7	459	0.6	1037	0.7	0.002
Malignancy or immunosuppression	44 757	20.6	15 553	20.8	29 204	20.5	0.092
Severe mental illness	4424	2.0	1358	1.8	3066	2.1	<0.001
Peripheral vascular disease	3645	1.7	1381	1.8	2264	1.6	<0.001
Stroke or transient ischaemic attack	10 834	5.0	3680	4.9	7154	5.0	0.316
Deaths within 28 days of positive COVID-19 test	5616	2.6	1476	2.0	4140	2.9	<0.001
Patients with at least one ED attendance within 28 days of positive COVID-19 test	43 250	19.9	9965	13.3	24 285	17.0	<0.001
Patients with at least one emergency admission within 28 days of positive COVID-19 test	26 529	12.2	7539	10.1	18 990	13.3	<0.001
Critical care use of those admitted	4275	16.1	1248	16.6	3027	15.9	0.220
Total	217 650		74 900	34.4	142 750	65.6	

CEV, clinically extremely vulnerable; CO@h, COVID Oximetry @home.

**Figure 2:**
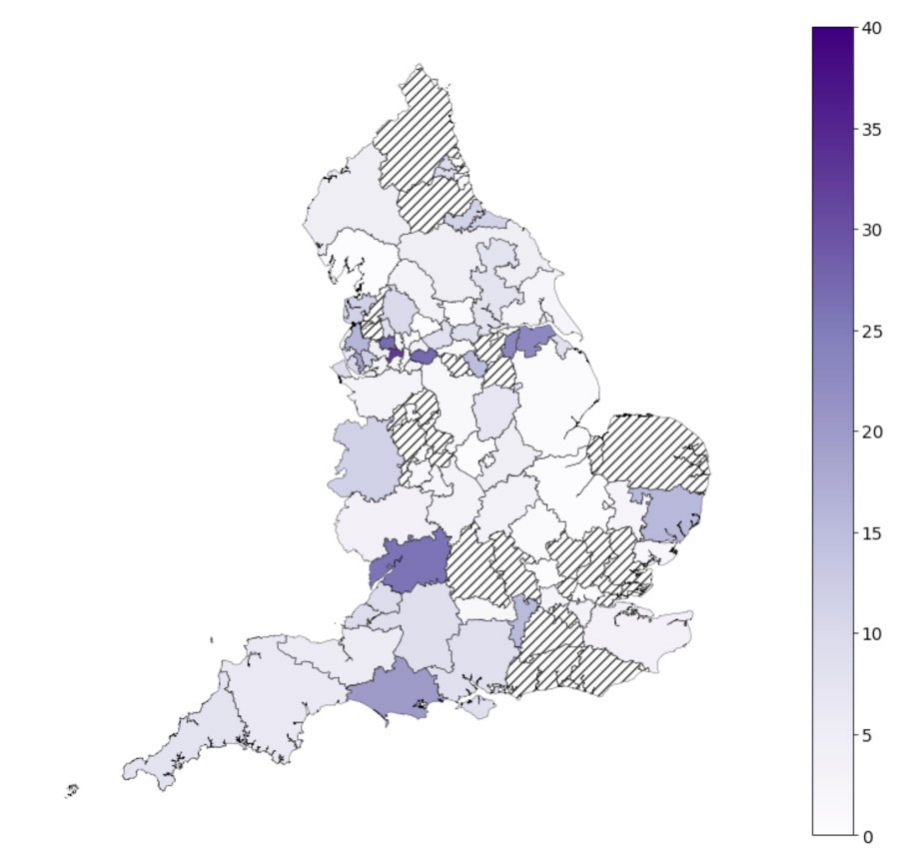
Percentage of the eligible population enrolled onto the COVID Oximetry @home programme in each Clinical Commissioning Group (CCG) from date of implementation based on submissions from sites. Hashed areas represent CCGs with no patient enrolment data submitted.

Data were received via submissions from CO@h sites for 5527 patients enrolled onto the programme, giving an overall enrolment rate based on submitted data of 2.5%. There was considerable variation in uptake across the 106 CCGs, ranging from 0.0% to 33.0% total enrolment, with a median of 2.2% (figure 2). The earliest date a CO@h site became operational was 20 November 2020, with all sites operational from 10 January 2021.

Mixed effects logistic regression was run separately for each outcome, with CCG of residence as a random intercept. Table 2 shows the results for the primary analysis for each outcome, adjusted for month of COVID-19 test and patient-level covariates. There was no significant difference in the adjusted odds of 28-day mortality in the period following implementation of the CO@h programme (adjusted OR (aOR)=1.06, p=0.405). There was evidence of a small increase in both ED attendances (aOR=1.12, p<0.001) and emergency hospital admissions (aOR=1.12, p<0.001) within 28 days. Of those patients admitted to hospital in the period after implementation, there was a 24% increase in the adjusted odds of requiring critical care (aOR=1.24, p=0.012). There was no significant difference in the length of stay of those admitted (p=0.588).

**Table 2:** Effect estimates for the implementation period of the CO@h programme, from mixed effects regression models, adjusted for month of test and patient-level covariates

Outcome	Adjusted OR	SE	P value	95% CI		Denominator
				Lower	Upper	
Mortality within 28 days of positive COVID-19 test	1.06	0.072	0.405	0.93	1.21	203 218
Any ED attendance within 28 days of positive COVID-19 test	1.12	0.033	<0.001	1.06	1.18	203 218
Any hospital admission within 28 days of positive COVID-19 test	1.12	0.037	<0.001	1.05	1.20	203 218
Critical care use of those admitted	1.24	0.107	0.012	1.05	1.47	24 895

	Adjusted IRR	SE	P value	Lower	Upper	Denominator
Length of stay of those admitted (in days)	1.02	0.029	0.588	0.96	1.07	20 794

Denominator represents the total number in the analysis with no missing data in adjusting covariates.

CO@h, COVID Oximetry @home; IRR, incidence rate ratio from negative binomial regression.

### Sensitivity analyses

Sensitivity analyses comparing alternative model specifications are given in the online supplemental tables A1–A5. Naïve models (unadjusted for time) showed significant increases in 28-day mortality, ED attendance and admissions associated with the programme and a weak association with lower odds of critical care admission. Little meaningful difference was seen between models unadjusted or adjusted for patient-level covariates, or with the addition of random time by CCG interactions. The intraclass coefficients for both CCG and CCG by time interactions for mortality, ED attendance and hospital admission models were all <1%, suggesting minimal variation between CCGs that might be accounted for by time-varying CCG factors.

### Secondary analysis of high enrolment CCGs

Secondary analyses were performed for 16 CCGs with 10% or more enrolment (table 3), and for 5 CCGs with 20% or more enrolment (table 4), representing 9.4% and 2.4% of the eligible population, respectively. In the 10% enrolment group, there was a 9% lower odds or mortality, 10% higher odds of ED attendance and 23% higher odds of critical care admission after implementation, but effects were statistically non-significant. There was evidence of 27% higher odds of admission (p=0.046). In the 20% enrolment group, effect sizes were larger, but none was statistically significant.

**Table 3:** Effect estimates for CO@h sites with 10% or more enrolment, from mixed effects logistic/negative binomial regression models adjusted for month of test and patient factors

Outcome	Adjusted OR	SE	P value	95% CI		Denominator
				Lower	Upper	
Mortality within 28 days of positive COVID-19 test	0.91	0.225	0.715	0.56	1.48	19 724
Any ED attendance within 28 days of positive COVID-19 test	1.10	0.120	0.369	0.89	1.37	19 724
Any hospital admission within 28 days of positive COVID-19 test	1.27	0.151	0.046	1.00	1.60	19 724
Critical care use of those admitted	1.23	0.373	0.496	0.68	2.23	2608

	Adjusted IRR	SE	P value	Lower	Upper	Denominator
Length of stay (days) of those admitted	1.13	0.112	0.213	0.93	1.37	2171

Denominator represents the total number in the analysis with no missing data in adjusting covariates.

CO@h, COVID Oximetry @home; IRR, incidence rate ratio.

**Table 4:** Effect estimates for CO@h sites with 20% or more enrolment, from mixed effects logistic/negative binomial regression models adjusted for month of test and patient factors

Outcome	Adjusted OR	SE	P value	95% CI		Denominator
				Lower	Upper	
Mortality within 28 days of positive COVID-19 test	0.89	0.397	0.798	0.37	2.13	4807*
Any ED attendance within 28 days of positive COVID-19 test	1.27	0.248	0.216	0.87	1.86	4887
Any hospital admission within 28 days of positive COVID-19 test	1.44	0.317	0.102	0.93	2.21	4887
Critical care use of those admitted	1.64	0.954	0.393	0.53	5.13	652

	Adjusted IRR	SE	P value	Lower	Upper	Denominator
Length of stay (days) of those admitted	1.27	0.259	0.246	0.85	1.89	552

Denominator represents the total number in the analysis with no missing data in adjusting covariates.

*80 observations excluded in April/May as no deaths occurring.

CO@h, COVID Oximetry @home; IRR, incidence rate ratio.

## Discussion

At a population level, there was no change in mortality in the period after implementation of the CO@h programme. The period of implementation was associated with statistically significant 12% increases in ED attendances and emergency hospital admissions and there was some evidence of an increase in the odds of receiving critical care in patients admitted. Although not statistically significant, the findings from the secondary analysis of high enrolment sites demonstrated a trend towards lower mortality and higher healthcare utilisation after the period of implementation.

Overall enrolment onto the programme was lower than expected, with data received from only 2.5% of the eligible population. This may be due to incomplete data submissions or may reflect genuinely low enrolment onto the programme and represents the lowest bound of true enrolment. If the 2.5% enrolment reflects the true number enrolled onto CO@h, then there is likely to be a dilutional effect on our population-level analysis and the effect estimates are likely to be picking up changes external to, rather than from, the programme itself.

A separate study using the same data sources showed significant increases in hospitalisation and fatality risk from COVID-19 over the time period CO@h sites were becoming operational.^20^ These trends may relate to winter effects on mortality, new treatments, hospital pressures, changes to admission criteria and the alpha COVID-19 variant which became the dominant strain in England during December 2020 and has been linked to significantly higher mortality compared with earlier variants.^21 22^ Introduction of the vaccination programme in December 2020 in England is likely to have had a protective effect on hospitalisation and mortality, particularly for higher-risk people, who were among the first eligible for vaccination.^23^ Despite our analysis incorporating time as an adjusting covariate, there may be residual confounding between sites becoming operational, and changes in the underlying hospitalisation and mortality rates.

The increases seen in ED attendances, emergency hospital admissions and critical care use following implementation of CO@h may reflect early recognition of silent hypoxaemia in COVID-19. The magnitude of increase in ED attendances was similar to the magnitude of increase in hospital admissions, suggesting that implementation did not cause a large increase in ED attendances not requiring admission. However, early intervention might be expected to decrease length of stay and mortality, which was not found here and could reflect changes to disease severity across the time of implementation.

Remote monitoring technologies have been widely used in the management chronic diseases, but with mixed evidence of their effectiveness^24^ and limited evidence for their use in COVID-19 with which to compare our findings.^25^ A pilot study of four NHS COVID-19 pulse oximetry programmes in England indicated the pathway was safe, but did not include a control group.^5^ A study in the USA of patients with COVID-19 referred to a remote patient monitoring pathway after discharge from hospital found lower odds of ED or hospital reattendance in those enrolled, but did not assess mortality.^26^

In a separate study of CO@h programme by the same authors, patients with COVID-19 enrolled to CO@h after assessment in ED had lower odds of mortality and critical care use, and higher odds of subsequent ED use and admission, compared with matched controls, although selection bias may limit the generalisability of the findings.^27^ Collectively, results from the two studies suggest that although there was no impact on mortality at a population level, there is some evidence for the effectiveness of the CO@h programme at an individual level (although within a narrower eligibility cohort of patients assessed in ED), but indicate that the programme could not be scaled up to provide a benefit to all eligible people nationally. Neither of the studies indicate that the programme causes harm, but it is unclear whether the results are generalisable to other forms of remote monitoring in COVID-19. There is a need for further research into patient and staff experience of the programme, and the barriers and facilitators to implementation of the programme which may explain the significant variation in enrolment across CCGs, which may aid policymakers and commissioners in implementing remote monitoring programmes in the future. There is also a need to understand the equity of access to CO@h, and to evaluate user experience and cost implications.

### Strengths and limitations

A strength of this study is the availability of comprehensive data on COVID-19 testing. Through linkage to primary and secondary care records, we were able to identify the eligible population and adjust for risk factors pre-implementation and post-implementation. Eligibility for the programme was not absolute, with a recommendation to extend to those aged 50 years and over from February 2021 and further variation across sites. Our analysis focused on a narrower subgroup of people aged 65 years or over or CEV, who would have remained eligible over the full study period, but findings might not be generalisable to all those included in the programme or to lower risk cohorts.

The stepped wedge design was chosen in part as it is robust to selection biases in enrolment of patients (eg, if there were systematic enrolment of patients with higher or lower risk or severity), which would impact individual-level study designs. The effect estimates are also not impacted by lack of submission of patient-level programme data on patients enrolled, which may not be complete. However, the fact we do not know whether the low numbers represent incomplete data or true enrolment impacts on our ability to judge if the study was adequately powered to detect a difference in mortality should one exist. Although our analysis accounted for underlying risk, we could not account for disease severity at diagnosis. Furthermore, some areas may have existing remote pulse oximetry services prior to the roll-out of the CO@h programme, which were either replaced or relabelled as CO@h, which may lead to a dilution of effect sizes.

Incorporation of additional CCG-level and hospital-level covariates, such as hospital bed and intensive care occupancy, was considered but estimates of the residual variation explained by clustering at the CCG-level (intraclass correlation) were small, suggesting these would have limited impact. Sensitivity analyses considered time-varying CCG-level effects, with almost identical results compared with the primary analysis, suggesting minimal impact of time-varying differences between CCGs. Across England, CO@h sites implemented different types of model, run by different sectors of the healthcare system, and with different recommendations for the frequency of monitoring.^4 8^ National population effect estimates as presented here may therefore mask variation in the effectiveness between sites. Our approach assumed that each site represents a discrete unit, but some sites may not cover an entire CCG, while others may provide services across boundaries.

## Conclusion

Implementation of the CO@h programme across England had no impact on mortality at a population level and was associated with small increases in ED attendances, hospitalisations and critical care use in people with COVID-19 aged 65 years or over or CEV, which may indicate early recognition of hypoxaemia and escalation. Lower than expected enrolment of eligible people may have diluted the effects of the programme at a population level. There is a need for further research into the uptake and effectiveness of remote monitoring programmes for COVID-19.

## Data Availability

Data may be obtained from a third party and are not publicly available. The patient-level data used in this study are not publicly available but are available to applicants meeting certain criteria through application of a Data Access Request Service (DARS) and approval from the Independent Group Advising on the Release of Data.
