# Supplementary material for: Population-level impact of a pulse oximetry remote monitoring programme on mortality and healthcare utilisation in the people with COVID-19 in England: a national analysis using a stepped wedge design
Source: Emerg Med J. 2022 Apr 12:emermed-2022-212378. doi: 10.1136/emermed-2022-212378 (PMC9023854; doi:10.1136/emermed-2022-212378)
Supplement: Supplementary file 1 [file emermed-2022-212378supp001.pdf]

Appendix

Population level impact of a pulse oximetry remote monitoring programme on mortality and healthcare utilisation in the people with COVID-19 in England: a national analysis using a stepped wedge design

Beaney T[1,2], Clarke J[1,3], Alboksmaty A[1,2], Flott K[1], Fowler A[4], Benger JR[5], Aylin P[1,2], Elkin S[6], Neves AL[1], Darzi A[1]

1. Patient Safety Translational Research Centre, Institute of Global Health Innovation, Imperial College London, London, SW7 2AZ, United Kingdom
2. Department of Primary Care and Public Health, Imperial College London, London, W6 8RP, United Kingdom
3. Centre for Mathematics of Precision Healthcare, Department of Mathematics, Imperial College London, London, SW7 2AZ, United Kingdom
4. NHS England and Improvement, London, SE1 6LH, United Kingdom
5. NHS Digital, 7-8 Wellington Place, Leeds, West Yorkshire, LS1 4AP, United Kingdom
6. National Heart and Lung Institute, Imperial College London, London, SW7 2AZ United Kingdom

Corresponding Author:
Dr Thomas Beaney
Patient Safety Translational Research Centre, Institute of Global Health Innovation, Imperial College London, London, SW7 2AZ, United Kingdom
Email: thomas.beaney@imperial.ac.uk

Entry criteria for COVID Oximetry @home

Eligibility for the COVID Oximetry @home (CO@h) programme is defined in the NHS England Standard Operating Procedure (SOP) although there was flexibility is operational models and eligibility across sites.[1]

Monitoring requirements could vary across sites, and involve text messages, email prompts or check-in calls, or support for full self-management and escalation.[1] Pulse oximeters were purchased by individual sites, or requested from the NHS Supply Chain and were required to meet ISO 80601-2-61:2017 and be CE marked.[1]

Data cleaning

Note: the described datasets were used for several distinct analyses by the study team, with data cleaning rules the same or similar between studies. For this reason, some of the text in the appendices may be identical to that of other published articles by the study authors on the same data source.

COVID-19 testing data

Testing data was provided through the Public Health England Second Generation Surveillance System (SGSS). This dataset captures routine laboratory data on infectious diseases for England, including COVID-19, with all diagnostic laboratories required to notify positive test results within 24 hours.[2] Data included 3,251,225 tests performed from 1st October 2020 to 30th June 2021, inclusive.

Provided data included test date and result date. 99% of results were reported within 5 days of the test, and 6,544 (0.2%) were reported more than 7 days from date of test. Where the result data occurred before the test date. In 1,603 cases, the result date was recorded as prior to the test date. In these instances, where the difference between the testing date and reporting date was 7 days or less, the test date and reporting date were swapped. In the 191 instances where reporting date was more than 7 days before the testing date, the test was excluded.

For this analysis, only tests performed up to 3rd May 2021 were included (after swapping test and result dates where applicable), given that secondary care data was available up until the end of May 2021. Of the 2,928,802 positive COVID-19 tests, 2,352,390 (80.3%) were from Pillar 2 testing, 561,852 (19.2%) from Pillar 1, and 14,560 (0.5%) from Pillar 4.[3] Test type was recorded for Pillar 2 tests only, and of these, 2,250,288 (95.7%) were Polymerase Chain Reaction (PCR) tests, 102,102 (4.3%) were lateral flow tests. Of PCR tests, 1,859,053 (82.6%) were documented as symptomatic at the time of test.

2,536,322 individuals had one or more positive COVID-19 tests. This analysis used only those with a positive PCR tests from pillar 2 testing, where symptoms were documented at the time of test, resulting in a total population of 1,799,547 people.

COVID Oximetry @home (CO@h) programme data

Data on patients enrolled ('onboarded') onto the CO@h programme were submitted directly from CO@h sites via NHS Digital's Strategic Data Collection Service.[4] Data included a deidentified NHS patient ID of the patient onboarded, along with the date of onboarding to and offboarding from the programme.

COVID Oximetry @home (CO@h) sites

Start dates at which each CO@h site became operational were provided by the NHS England @home programme at a Clinical Commissioning Group (CCG) level. Start dates were available for all CCGs in England, except for NHS commissioning hubs, which were excluded (National Commissioning Hub 1 (code 13Q), London Commissioning Hub (code 13R) and National Commissioning Hub 2 (code 15L)). The CCG assignments as of April 2021 were used, and in cases where CCGs had merged, the earliest start date of the programme in the merged CCGs was used. The population was assigned to the CCG recorded in the SGSS dataset at the time of their test, and those outside of England were excluded. A final eligible cohort of 1,714,182 individuals with a PCR positive, and symptomatic at the time of test, were included

Shielded Patient List

Identification of high-risk patients, designated 'Clinically Extremely Vulnerable' (CEV) was provided via a linkage from the NHS Digital Shielded Patient List (SPL) to the primary care record.[5] Patients with any of the conditions listed in Box A1 below were designated as high risk.[6] Code lists for the conditions are available to download from NHS Digital.[7] In addition, from February 2021, people were also added to the list if identified as high risk using the QCovid risk prediction model, which combines factors including age, sex, ethnicity, BMI as well as specific conditions.[8] Thresholds of mortality risk for inclusion in the SPL were an absolute risk of 0.5% or higher, or a relative risk of 10 times the baseline risk of a person with the same age and sex.[6] GP practices and NHS Trusts were also able to add and remove patients from the SPL based on clinical judgment and individual risk assessments.[9]

Box A1: NHS Digital criteria for Clinically Extremely Vulnerable patients:

- solid organ transplant recipients
- people with severe respiratory conditions including all cystic fibrosis, severe asthma and severe chronic obstructive pulmonary (COPD)
- people with rare diseases and inborn errors of metabolism that significantly increase the risk of infections (such as Severe combined immunodeficiency (SCID), homozygous sickle cell)
- people on immunosuppression therapies sufficient to significantly increase risk of infection
- people who have problems with their spleen, for example have had a splenectomy
- adults with Down's syndrome
- adults on dialysis with kidney impairment (Stage 5 Chronic Kidney Disease)
- women who are pregnant with significant heart disease, congenital or acquired
- people with cancer who are undergoing active chemotherapy
- people with lung cancer who are undergoing radical radiotherapy
- people with cancers of the blood or bone marrow such as leukaemia, lymphoma or myeloma who are at any stage of treatment
- people having immunotherapy or other continuing antibody treatments for cancer
- people having other targeted cancer treatments which can affect the immune system, such as protein kinase inhibitors or PARP inhibitors
- people who have had bone marrow or stem cell transplants in the last 6 months, or who are still taking immunosuppression drugs

Source: https://digital.nhs.uk/coronavirus/shielded-patient-list/risk-criteria

Primary care data

Primary care data came from the General Practice Extraction Service (GPES) Data for Pandemic Planning and Research (GDPPR).[10] Data included month and year of birth, sex, ethnicity, Lower Layer Super Output Area (LSOA) of residence, a marker for CEV status (linked from SPL) , and a marker for residence in a care home. LSOA was used to link to 2019 deciles of Index of Multiple Deprivation (IMD).[11] Age was calculated from date of positive COVID-19 test, assuming a birthdate on the 15th day of the month.

Entries include a date to which each journal item applies, and a date on which the journal item was recorded. The former was used in priority, but where missing, was replaced with the journal item recording date. For LSOA, CEV status and care home residence, only entries occurring up to the date of positive COVID-19 test were included. For month and year of birth, sex and ethnicity, if no entry were included prior to the date of COVID-19 test, then the earliest recorded entry after the test was included.

Secondary care data

Data on hospital admissions came from the Hospital Episode Statistics (HES) data set up to 31st May 2021, linked to Office for National Statistics (ONS) data on death registrations up to 5th July 2021.[12] Entries were excluded where missing admission dates, provider Trust code, or patient deidentified ID.

Where multiple admission episodes were recorded within a spell, a single spell start and end date were created. Non-emergency hospital admissions were excluded from analyses. A binary indicator was created for any admission within 28 days of positive COVID-19 test. A second indicator was created for death (of any cause) within 28 days of positive COVID-19 test. Critical care admissions were defined as an admission episode for any level 2 or level 3 care within the defined spell.

Data on accident and emergency (A&E) attendances came from the Emergency Care Data Set (ECDS), including both Type 1 (24-hour consultant led A&E departments) and Type 2 (Specialist Emergency Departments).[13] The A&E attendance date, departure date and admission date (if subsequently admitted) are included. In cases where attendance date was recorded as being after the departure date, the attendance date was set to the departure date, if

departure date was equal to the admission date. Otherwise, attendance date was assumed to be correct.

Where multiple A&E attendances were recorded on the same day, a single attendance was kept for each patient, prioritising in turn:

1. Any attendance associated with an admission
2. Earliest time of attendance
3. Earliest time of departure

A binary indicator was created for one or more A&E attendances within 28 days of a positive COVID-19 test.

Where age was missing from GDPPR, it was derived from month and year of birth in HES, or if also missing in HES, derived from month and year of birth in ECDS, using the same approach as for GDPPR. Where LSOA was missing from GDPPR, it was derived from HES/ECDS. CCG was derived first from testing data, and if missing, from CO@h programme data, followed by, in order of use, GDPPR, HES or ECDS.

Co-morbidities

SNOMED codes were included in the GDPPR dataset pertaining to specific SNOMED code cluster reference sets provided by NHS Digital.[10] 6,485 unique codes were identified from GDPPR. Codes were reviewed manually by authors TB and JC and removed if not relevant or assigned to the minimal number of relevant code clusters.

SNOMED reference clusters were aggregated into hierarchies of similar conditions. Codes in each higher-order cluster were then reviewed to ensure groupings of relevant codes and twelve relevant chronic disease categories were selected: hypertension, chronic cardiac disease, chronic kidney disease, chronic respiratory disease, dementia, diabetes, chronic neurological disease (including epilepsy), learning disability, malignancy/immunosuppression, severe mental illness, peripheral vascular disease and stroke/transient ischaemic attack (TIA). Categories for chronic respiratory disease, diabetes, epilepsy, malignancy/immunosuppression and severe mental illness included relevant medication codes. Broad diagnostic categories of diagnoses were chosen, as certain

medications were not diagnostic of more granular diagnostic categories (for example, use of a long-acting bronchodilator/inhaled corticosteroid in both COPD and asthma).

For each patient in GDPPR, all relevant diagnostic codes prior to the study index date (date of positive COVID-19 test) were considered diagnostic. In cases where the latest SNOMED code indicated resolution of a condition (e.g. 'Atrial fibrillation resolved (finding)'), then the diagnosis was excluded for that patient. SNOMED codes relating to drug codes were only included up to 2 years prior to the index date.

A full list of codes within each diagnostic category are available in our GitHub repository: https://github.com/tbeaney/Imperial-COh-evaluation

BMI categorisation

SNOMED codes for BMI were either diagnostic categories (eg 'Body mass index 30+ - obesity (finding)' or value codes (e.g. 'Body mass index (observable entity)'). Values were extracted and BMI was categorised according to the standard World Health Organisation classification of underweight (<18.5 kg/m^2), healthy weight (18.5-24.9 kg/m^2), overweight (25.0-29.9 kg/m^2) and obese (≥30.0 kg/m^2). Value codes outside of the range 5.0-100.0 kg/m2 were excluded. SNOMED codes which spanned more than one category (e.g. 'Increased body mass index (finding)') and child BMI categories were also excluded.

Smoking categorisation

Smoking status was categorised into 'never-smoker', 'ex-smoker' and 'current smoker' according to the latest SNOMED code prior to and including the index date. For any patient where the latest SNOMED code indicated 'never-smoker', but a prior record indicated active smoking, then the patient was re-categorised as 'ex-smoker.

Statistical analysis

Sensitivity analyses

To test the sensitivity of results to time-varying effects and differences in patient risk factors, a series of sensitivity analyses were run:

1. A naïve model, not accounting for time or patient-level covariates
2. A time-adjusted model, without adjusting for patient-level covariates
3. A time-adjusted model, incorporating a random interaction between time and CCG
4. A time-adjusted model incorporating a random interaction between time and CCG, and adjusting for patient-level covariates

Table A1: Effect sizes for the CO@h programme on 28-day mortality from mixed effects logistic regression models under different model specifications (n=217,650)

Model	Odds ratio	Standard error	p-value	95% confidence interval		Denominator*
				Lower	Upper	
Naïve	1.53	0.05	<0.001	1.44	1.63	217,650
Time adjusted models						
Simple	1.07	0.068	0.259	0.95	1.22	217,650
Full adjustment for patient factors	1.06	0.072	0.405	0.93	1.21	203,218
Random time by CCG interaction						
Simple	1.07	0.069	0.291	0.95	1.22	217,650
Full adjustment for patient factors	1.06	0.072	0.407	0.93	1.21	203,218

*Denominators lower in adjusted models due to missingness in patient-level covariates

Table A2: Effect sizes for the CO@h programme on any A&E attendance within 28 days from mixed effects logistic regression models under different model specifications (n=217,650)

Model	Odds ratio	Standard error	p-value	95% confidence interval		Denominator*
				Lower	Upper	
Naïve	1.31	0.018	<0.001	1.27	1.34	217,650
Time adjusted models						
Simple	1.13	0.032	<0.001	1.07	1.19	217,650
Full adjustment for patient factors	1.12	0.033	<0.001	1.06	1.18	203,218
Random time by CCG interaction						
Simple	1.12	0.033	<0.001	1.06	1.19	217,650
Full adjustment for patient factors	1.11	0.035	0.001	1.04	1.18	203,218

*Denominators lower in adjusted models due to missingness in patient-level covariates

Table A3: Effect sizes for the CO@h programme on any hospital admission within 28 days from mixed effects logistic regression models under different model specifications (n=217,650)

Model	Odds ratio	Standard error	p-value	95% confidence interval		Denominator*
				Lower	Upper	
Naïve	1.39	0.021	<0.001	1.35	1.43	217,650
Time adjusted models						
Simple	1.13	0.035	<0.001	1.07	1.20	217,650
Full adjustment for patient factors	1.12	0.037	<0.001	1.05	1.20	203,218
Random time by CCG interaction						
Simple	1.13	0.036	<0.001	1.06	1.20	217,650
Full adjustment for patient factors	1.12	0.038	0.001	1.05	1.20	203,218

*Denominators lower in adjusted models due to missingness in patient-level covariates

Table A4: Effect sizes for the CO@h programme on any critical care admission of those admitted from mixed effects logistic regression models under different model specifications (n=26,529)

Model	Odds ratio	Standard error	p-value	95% confidence interval		Denominator*
				Lower	Upper	
Naïve	0.92	0.036	0.04	0.85	1.00	26,529
Time adjusted models						
Simple	1.15	0.093	0.087	0.98	1.35	26,529
Full adjustment for patient factors	1.24	0.107	0.012	1.05	1.47	24,895
Random time by CCG interaction						
Simple	1.13	0.094	0.148	0.96	1.33	26,529
Full adjustment for patient factors	1.23	0.109	0.019	1.04	1.46	24,895

*Denominators lower in adjusted models due to missingness in patient-level covariates

Table A5: Effect sizes for the CO@h programme on length of stay of those admitted from mixed effects negative binomial regression models under different model specifications (n=26,529)

Model	Incidence rate ratio	Standard error	p-value	95% confidence interval		Denominator*
				Lower	Upper	
Naïve	1.04	0.015	0.004	1.01	1.07	22,079
Time adjusted models						
Simple	1.02	0.029	0.495	0.96	1.08	22,079
Full adjustment for patient factors	1.02	0.029	0.588	0.96	1.07	20,794
Random time by CCG interaction						
Simple	1.02	0.029	0.455	0.97	1.08	22,079
Full adjustment for patient factors	1.02	0.029	0.588	0.96	1.07	20,794

*Denominators lower in adjusted models due to missingness in patient-level covariates

References

1. NHS England and Improvement. Novel coronavirus (COVID-19) standard operating procedure: COVID Oximetry @home. (2021).
2. Public Health England. *Laboratory reporting to Public Health England: A guide for diagnostic laboratories*. (2020).
3. Department of Health & Social Care. COVID-19 testing data: methodology note. https://www.gov.uk/government/publications/coronavirus-covid-19-testing-data-methodology/covid-19-testing-data-methodology-note (2021).
4. NHS Digital. Strategic Data Collection Service (SDCS). https://digital.nhs.uk/services/strategic-data-collection-service-sdcs.
5. NHS Digital. Shielded Patient List. https://digital.nhs.uk/coronavirus/shielded-patient-list.
6. NHS Digital. Shielded Patient List Risk Criteria. https://digital.nhs.uk/coronavirus/shielded-patient-list/risk-criteria (2021).
7. NHS Digital. Shielded Patient List: Annexes. https://digital.nhs.uk/coronavirus/shielded-patient-list/methodology/annexes.
8. NHS Digital. Shielded Patient List: COVID-19 Population Risk Assessment. https://digital.nhs.uk/coronavirus/risk-assessment/population.
9. NHS Digital. Guidance for clinicians about the COVID-19 Clinical Risk Assessment Tool. https://digital.nhs.uk/coronavirus/risk-assessment/clinical-tool/guidance-for-clinicians.
10. NHS Digital. General Practice Extraction Service (GPES) Data for pandemic planning and research: a guide for analysts and users of the data. https://digital.nhs.uk/coronavirus/gpes-data-for-pandemic-planning-and-research/guide-for-analysts-and-users-of-the-data (2021).
11. Ministry of Housing & Communities & Local Government. English indices of deprivation 2019. https://www.gov.uk/government/statistics/english-indices-of-deprivation-2019.
12. Digital, N. Hospital Episode Statistics (HES). https://digital.nhs.uk/data-and-information/data-tools-and-services/data-services/hospital-episode-statistics.
13. NHS Digital. Emergency Care Data Set (ECDS). https://digital.nhs.uk/data-and-information/data-collections-and-data-sets/data-sets/emergency-care-data-set-ecds.
